# Integrated tear proteomics define the molecular blueprint of corneal epithelial repair

**DOI:** 10.3389/ebm.2025.10866

**Published:** 2026-02-02

**Authors:** Nadege Feret, Marilou Decoudu, Jerome Vialaret, Christophe Hirtz, Karine Loulier, Vincent Daien, Frederic Michon

**Affiliations:** 1 Institute for Neurosciences of Montpellier, University Montpellier, INSERM, Montpellier, France; 2 IRMB-PPC, University Montpellier, CHU Montpellier, INSERM CNRS, Montpellier, France; 3 Department of Ophthalmology, Gui de Chauliac Hospital, Montpellier, France; 4 The Save Sight Institute, Sydney Medical School, The University of Sydney, Sydney, NSW, Australia

**Keywords:** cornea, epithelium, human, mouse, proteomic

## Abstract

Tears are easy to collect, repeatable, and reflect the state of the corneal surface—attributes that make them attractive for bedside monitoring after surgery or injury. We performed a cross-species meta-analysis of tear proteomes from patients undergoing photorefractive keratectomy (PRK) and from mice after mechanical epithelial abrasion to define molecular programs that are both conserved and clinically actionable. Roughly one-third of the injury response was shared between species, centering on innate immune activation (complement/acute phase), epithelial migration and cytoskeletal remodeling, and a calibrated suppression of proteolysis. From this overlap we distilled a small, secreted tear panel that stages injury and early resolution in both species: transferrin and hemopexin (iron/heme scavenging), albumin (vascular leak), apolipoprotein A-I (barrier lipid transport), and the coagulation modulators kininogen-1 and α2-antiplasmin (protease/fibrinolysis control). This panel rises at the first post-injury sampling (D0 in humans; 6–12 h in mice) and trends toward baseline during recovery (D3 in humans; ∼24 h in mice), providing a practical kinetic signature for clinical decision-making. Standardized sampling at D0/D3 can therefore quantify acute damage and early healing, enable pharmacodynamic readouts for anti-inflammatory or barrier-stabilizing therapies, and support risk stratification after epithelial procedures. Species-specific differences (human: secretory/immune surveillance; mouse: mitochondrial/metabolic reboot) clarify which preclinical signals are most likely to translate. Together, these findings establish a conserved tear blueprint of corneal repair and nominate a minimal, deployable biomarker set to accelerate clinical monitoring and therapeutic development in ocular surface disease.

## Impact statement

Our study shows that the healing of the eye’s surface follows a shared, measurable pattern in both humans and mice, and that this pattern can be tracked non-invasively in tears. By aligning two independent tear datasets, we identify a small set of tear proteins that reliably rise after injury and then return toward normal as healing proceeds. This work is important because it turns a complex, hard-to-monitor process into simple readouts that can be collected at the bedside. It advances the field by bridging animal and human findings and by offering practical markers to stage injury and recovery. The new information is a conserved healing signature and a minimal marker panel with clear timing. This impacts the field by enabling better monitoring after eye procedures and providing ready-to-use tools for future treatment trials.

## Introduction

The vertebrate cornea represents a highly conserved anatomical and physiological structure across diverse species, characterized by transparency, avascularity, and a meticulously organized extracellular matrix crucial for optimal visual performance [1, 2]. Despite substantial evolutionary divergence among vertebrates, the fundamental architecture of the cornea—comprising the epithelium, stroma, and endothelium—remains remarkably consistent [3]. This structural conservation highlights strong evolutionary pressures aimed at preserving corneal transparency and integrity, both essential for vision-dependent behaviors such as predation, navigation, and reproduction [1].

The evolutionary transition from aquatic to terrestrial life necessitated significant physiological adaptations, among which the development of the tear film stands prominent. This multilayered fluid interface is crucial for maintaining corneal hydration, providing trophic support, offering protection against pathogens, and preserving optical clarity [4]. The emergence of the tear film is tightly coupled to terrestrial adaptation, acting as a defense mechanism against new environmental stressors such as desiccation, mechanical damage, and microbial threats, all of which intensified upon terrestrial colonization. Unlike aquatic ancestors whose corneal hydration was inherently supported by their aquatic surroundings, terrestrial vertebrates evolved this sophisticated tear film to actively maintain ocular surface homeostasis [5, 6].

Remarkably, across terrestrial vertebrate species, the tear film exhibits strikingly conserved structural and functional characteristics. It consists of three distinct yet integrated layers: a superficial lipid layer, an intermediate aqueous layer, and an inner mucinous layer [7]. The lipid layer, predominantly produced by the meibomian glands, prevents evaporation and stabilizes the ocular surface. The aqueous layer, secreted by lacrimal glands, delivers essential nutrients, growth factors, oxygen, and antimicrobial molecules. The mucinous layer, primarily produced by conjunctival goblet cells, ensures stable adhesion of the tear film to the hydrophobic corneal epithelium. The persistent conservation of this trilaminar structure emphasizes its evolutionary robustness as an effective physiological response to terrestrial environmental pressures [6].

The integrity and functionality of the tear film are closely intertwined with overall corneal health. Disruptions in tear film composition or stability underpin various ocular surface disorders, including dry eye syndrome, corneal ulcers, and neurotrophic keratitis [4, 8, 9]. Therefore, uncovering novel therapeutic targets within the tear film could profoundly impact clinical strategies, enhancing corneal healing processes and preserving vision [10, 11]. Achieving these therapeutic advances requires an in-depth understanding of tear film dynamics, particularly the molecular alterations occurring in response to corneal injury and may yield clinically useful biomarker signatures [12, 13].

To address this hypothesis, we performed a comparative analysis of tear film proteomic changes during corneal wound healing in murine and human models [14]. We leveraged two pivotal datasets generated in our recent studies: one describing murine corneal healing after physical abrasion [15], and the other detailing human corneal recovery following photorefractive keratectomy (PRK) [16]. Both proteomic datasets are publicly available (ProteomeXchange identifiers PXD061492, PXD067691), and were obtained on the same mass-spectrometry machinerie, facilitating a direct comparative approach. By re-analyzing these data, we identified conserved molecular mediators that constitute the fundamental response of the tear film to corneal injury. Elucidating this shared molecular framework holds promise for identifying universal therapeutic targets that enhance corneal repair across species and accelerate translational developments for ocular surface diseases by leveraging a scalable, standardized LC-MS/MS workflow tailored to tear fluid [17, 18].

By identifying these fundamental molecular signatures, our study aims to pinpoint core therapeutic targets with broad applicability across species. Such comparative investigations are expected to expedite the development of innovative therapeutic interventions, offering significant translational potential and paving the way for future research in ocular surface disease management.

## Materials and methods

### Cohorts characteristics

We extracted the lists of proteins identified in the murine [15] and human [16] tear film during corneal wound healing. For the human tear film analysis, the cohort was composed of 10 patients, respectively 3 men and 7 women, within a range from 24 to 36 years old (mean *± SD:* 30 *± 4.055). The PRK procedure was performed on both eyes and involved an epithelial removal step (abrasion) followed by stromal laser ablation to correct the refractive defect. Tear samples were collected separately from both eyes using Schirmer strips before the PRK surgery, less than 1 h after the surgery and 3 days post-surgery.* For the murine tear film analysis, the cohort was composed of 7 female Swiss/CD1 mice (RjOrl:SWISS, Janvier Labs, France), aged between 11 and 12 weeks old. The epithelial abrasion was performed unilateraly. The stroma was not wounded during this procedure. Tear samples were collected separately from both eyes using microcapillary glass tubes before the abrasion, 6, 12, 18 and 24 h post-abrasion.

### Analysis strategy

From the proteomics datasets, we selected the proteins exhibiting a fold change <0.5 or >2, and significantly dysregulated (p-value <0.05). We identified the proteins which were identified in the murine and human tear film.

For the integrative analysis, we first compiled the union of all proteins that reached statistical significance in at least one time point in at least one dataset. We then defined increasingly stringent subsets by applying more conservative filters to this union. In particular, for biomarker screening we selected proteins that (i) showed consistent direction of regulation across relevant time points and species, and (ii) exhibited a nominal p-value <0.001 together with an absolute fold-change >2.75 in the primary human dataset. These stricter thresholds were chosen to increase specificity and robustness of candidate markers in a meta-analytic context, by focusing on large-effect proteins with highly significant changes that are more likely to be reproducible across cohorts and analytical platforms and technically accessible in small tear volumes.

### Functional and pathway enrichment analyses

For each dataset, species and time point, we first identified differentially abundant proteins as those showing an absolute fold-change ≥2 (enriched) or ≤0.5 (depleted) together with a nominal p-value <0.05 in the corresponding post-injury versus baseline comparison. These proteins were taken forward for functional analyses, while all quantified proteins in the dataset were retained as the background universe for interpretation.

Enrichment analyses were performed using the Enrichr platform^1^. Protein identifiers were converted to official gene symbols prior to upload. Up-regulated and down-regulated protein sets were analysed separately. For each list, we queried the following gene-set libraries: Reactome Pathways, Gene Ontology (GO) Biological Process, GO Cellular Component, and GO Molecular Function. Enrichr computes enrichment using Fisher’s exact test and reports, for each term, a nominal p-value, an adjusted p-value based on the Benjamini–Hochberg false discovery rate (FDR) and a combined score that integrates the p-value with the deviation from the expected rank. We considered terms with FDR <0.05 and at least three contributing proteins as significantly enriched and used the combined score to rank pathways within each comparison.

To facilitate cross-timepoint and cross-species interpretation, the resulting enrichment profiles were visualised using Appyter^2^. Enriched terms from Reactome and the three GO ontologies were then manually curated into broader biological themes (immune/secretory surveillance, epithelial repair, extracellular-matrix remodelling, mitochondrial and metabolic programs) that are reported here.

## Results

### Description of the tear film composition modulation after corneal wounding

Tear film samples were collected immediately and 3 days following photorefractive keratectomy (PRK) in patients [16], and at 6, 12, 18, and 24 h after mechanical corneal abrasion in mice [15]. The tear proteomic profiles were analyzed by mass spectrometry (Supplementary Tables S1, S2). We considered all proteomic modulations occurring within these timeframes as indicative of the corneal injury response. The detailed comparative analysis of human and murine proteomic responses is summarized in Table 1, and the list of proteins is in Supplementary Table S3. In total, 2025 proteins were identified in human tear samples, while murine samples revealed 3,618 proteins. Notably, a similar number of proteins were found to be upregulated in both species (human: 667; mouse: 589). In contrast, the number of downregulated proteins in murine tears (640 proteins) was approximately three-fold higher than in human tears (242 proteins). Consequently, due to the overall lower number of proteins detected in human samples, the proportion of proteins modulated in response to injury was higher in humans. Interestingly, the human tear film response predominantly involved protein upregulation (representing 73.38% of the total injury response), whereas the murine response showed a balanced pattern, with approximately equal proportions of upregulated and downregulated proteins. Finally, a comparative analysis of the protein identities revealed that around one-third of the injury-associated proteomic response was conserved between mice and humans. This substantial overlap underscores a highly conserved core response, alongside significant species-specific proteomic adaptations.

**TABLE 1:** Overview of the corneal injury response in human and mouse.

Analysis	Human corneal injury response		Murine corneal injury response	
	Upregulated	Downregulated	Upregulated	Downregulated
Number of proteins / total number of identified proteins	667 / 2025	242 / 2025	589 / 3618	640 / 3618
Percentage of the tear proteome	32,94	11,95	16,28	17,69
Percentage of the injury response	73,38	26,62	47,93	52,07
Percentage of conservation of the injury response	26,99	9,50	30,56	3,59

### Conservation of the identified proteins

We identified 180 proteins that were upregulated and 23 proteins that were downregulated during the corneal injury response in both human and murine models (Supplementary Table S4). To elucidate the functional significance of these differentially expressed proteins, we conducted comprehensive enrichment analyses using Reactome, GO Biological Process, and GO Molecular Function databases, aiming to reveal crucial biological pathways significantly modulated during corneal wound healing (Table 2; Supplementary Tables S5–S10).

**TABLE 2:** Top 5 *Reactome Pathways*, *GO Biological Process, GO Molecular Functions* terms regulated by the proteins regulated similarly in human and mouse during corneal wound healing.

Analysis		*Term*	*p-value*	*q-value*
Reactome pathways	Upregulated	Eukaryotic translation elongation	*3,27E-91*	*1,93E-88*
		Nonsense mediated decay (NMD) independent of the exon junction complex (EJC)	*7,38E-91*	*2,03E-88*
		Peptide chain elongation	*1,03E-90*	*2,03E-88*
		Viral mRNA translation	*3,63E-90*	*5,37E-88*
		SRP-dependent cotranslational protein targeting to membrane	*2,43E-89*	*2,49E-87*
	Downregulated	Synthesis of UDP-N-acetyl-glucosamine	*3,53E-05*	*4,20E-03*
		Cytosolic sulfonation of small molecules	*3,44E-04*	*2,05E-02*
		Synthesis of substrates in N-glycan biosythesis	*2,44E-03*	*8,68E-02*
		Biosynthesis of the N-glycan precursor (dolichol LLO) and transfer to a nascent protein	*3,69E-03*	*8,68E-02*
		Asparagine N-linked glycosylation	*5,01E-03*	*8,68E-02*
GO biological process	Upregulated	Cytoplasmic translation (GO:0002181)	*1,71E-81*	*2,14E-78*
		Macromolecule biosynthetic process (GO:0009059)	*1,76E-67*	*1,10E-64*
		Translation (GO:0006412)	*9,85E-64*	*4,11E-61*
		Gene expression (GO:0010467)	*8,51E-60*	*2,66E-57*
		Protein metabolic process (GO:0019538)	*2,07E-44*	*5,18E-42*
	Downregulated	UDP-N-acetylglucosamine biosynthetic process (GO:0006048)	*3,53E-05*	*3,18E-03*
		Purine ribonucleotide metabolic process (GO:0009150)	*3,93E-05*	*3,18E-03*
		UDP-N-acetylglucosamine metabolic process (GO:0006047)	*5,66E-05*	*3,18E-03*
		Amino sugar biosynthetic process (GO:0046349)	*6,91E-05*	*3,18E-03*
		Cellular response to alkaloid (GO:0071312)	*1,50E-04*	*5,53E-03*
GO molecular function	Upregulated	mRNA binding (GO:0003729)	*5,89E-18*	*1,20E-15*
		Cadherin binding (GO:0045296)	*9,78E-12*	*9,98E-10*
		mRNA 5′-UTR binding (GO:0048027)	*6,47E-10*	*4,40E-08*
		rRNA binding (GO:0019843)	*4,08E-09*	*2,08E-07*
		Ubiquitin ligase inhibitor activity (GO:1990948)	*1,36E-08*	*5,54E-07*
	Downregulated	Intramolecular oxidoreductase activity interconverting aldoses and ketoses (GO:0016861)	*5,74E-03*	*7,68E-02*
		Protein geranylgeranyltransferase activity (GO:0004661)	*5,74E-03*	*7,68E-02*
		Nucleoside kinase activity (GO:0019206)	*6,88E-03*	*7,68E-02*
		Intramolecular phosphotransferase activity (GO:0016868)	*9,16E-03*	*7,68E-02*
		Cysteine-type peptidase activity (GO:0008234)	*1,23E-02*	*7,68E-02*

Our analysis highlighted a predominant upregulation of molecular pathways associated with protein synthesis and translation, specifically eukaryotic translation elongation, nonsense-mediated decay, and viral mRNA translation. Furthermore, the most significantly modulated GO biological processes and molecular functions pointed towards extensive cellular remodeling, adhesion dynamics, and protein regulation, indicating active roles in tissue restructuring and repair during corneal healing, as shown in Table 2.

Additionally, pathways recognized for their roles in corneal repair, such as axon guidance, keratinization, and Notch signaling activity, were notably upregulated (Supplementary Table S5). Processes critical to effective wound closure, including cell motility (Supplementary Table S6) and fibroblast growth factor (FGF) binding (Supplementary Table S7), also demonstrated significant elevation. Conversely, the limited number of downregulated factors led to suppression of pathways related to axonal growth inhibition (Supplementary Table S8), epithelial cell-cell adhesion (Supplementary Table S9), and actin binding (Supplementary Table S10)—processes essential for the dynamic rearrangement of epithelial cells during wound healing.

While this targeted analysis effectively identified essential biological processes in corneal wound healing, we recognized its inherent limitations due to the complexity and coordination of biological pathways. Therefore, we expanded our investigation by directly analyzing the reactome pathways, GO biological processes, GO cellular components and GO molecular functions modulated in the original human and mouse proteomic datasets to achieve a more holistic understanding of the corneal wound healing response.

### Conservation of integrative functions during corneal wound healing

We extracted the proteins differentially regulated during corneal wound healing in the human (909 proteins identified) and murine (1,229 proteins identified) tear film. Then, we analyzed the various integrative functions associated with these factors.

To identify conserved biological processes driving corneal wound healing, we performed pathway enrichment analyses using Reactome and Gene Ontology (GO) databases, focusing on the tear proteome profiles in both human and murine models. We specifically extracted shared upregulated and downregulated terms during the healing response to determine the core molecular programs mobilized during epithelial injury. The results reveal a highly coordinated and evolutionarily preserved response involving innate immune activation, cytoskeletal remodeling, controlled proteolysis, and metabolic reprioritization.

Among the Reactome pathways, complement activation emerged as a dominant feature, with strong upregulation of “Activation of C3 and C5” and “Alternative Complement Activation,” as presented in Table 3 (Supplementary Table S11). These cascades are central to the innate immune defense, mediating pathogen clearance, opsonization, and the recruitment of immune effectors to the injury site. The tear film appears to function as a first-line immune barrier, rapidly responding to epithelial breaches. Notably, this is accompanied by upregulation of the “Acute Inflammatory Response” and “Acute-Phase Response” GO terms, further supporting the role of the tear proteome in delivering immune mediators such as complement proteins, cytokines, and antimicrobial peptides directly to the ocular surface.

**TABLE 3:** Top 10 *Reactome Pathways* terms regulated similarly in human and mouse during corneal wound healing.

Reactome analysis		*Term*
Reactome pathways	Upregulated	L13a-mediated translational silencing of ceruloplasmin expression
		Formation of a pool of free 40S subunits
		GTP hydrolysis and joining of the 60S ribosomal subunit
		Cap-dependent translation initiation
		Eukaryotic translation initiation
		Eukaryotic translation elongation
		Peptide chain elongation
		Nonsense mediated decay (NMD) independent of the exon junction complex (EJC)
		Response of EIF2AK4 (GCN2) to amino acid deficiency
		Viral mRNA translation
	Downregulated	Parasite infection
		FCGR3A-mediated phagocytosis
		Leishmania phagocytosis
		Regulation of actin dynamics for phagocytic cup formation
		FCERI mediated NF-kB activation
		Fcgamma receptor (FCGR) dependent phagocytosis
		Fc epsilon receptor (FCERI) signaling
		Leishmania infection
		Parasitic infection pathways
		Signaling by the B Cell receptor (BCR)

Interestingly, while innate immune pathways were activated, several components of the adaptive immune system, including “MHC Class II Antigen Presentation” and “Fc Receptor Signaling,” were consistently downregulated. This suggests an intentional suppression of adaptive immune mechanisms during the acute wound healing phase, likely to prevent overactivation and subsequent tissue damage. Similarly, Reactome terms related to immunoglobulin glycosylation and antigen processing were also reduced. Such selective immune modulation highlights the unique requirement of the cornea to balance immune defense with the preservation of optical clarity.

In parallel with immune activation, we observed a robust upregulation of pathways associated with cytoskeletal dynamics. GO terms such as “Actin Filament Organization,” “Actomyosin Structure Organization,” and “Adherens Junction” were prominently enriched, as indicated in Table 4 (Supplementary Table S12). These processes are central to epithelial cell migration, a critical step in resurfacing the denuded corneal surface. The upregulation of actin and cadherin binding molecular functions further supports a program of collective epithelial cell movement, coordinated by adherens junction remodeling and actomyosin contractility.

**TABLE 4:** Top 10 *GO Biological Process* terms regulated similarly in human and mouse during corneal wound healing.

GO Analysis		*Term*
GO biological process	Upregulated	Cytoplasmic translation (GO:0002181)
		Macromolecule biosynthetic process (GO:0009059)
		Translation (GO:0006412)
		Gene expression (GO:0010467)
		Protein metabolic process (GO:0019538)
		RNA processing (GO:0006396)
		Protein-RNA complex assembly (GO:0022618)
		mRNA splicing, via spliceosome (GO:0000398)
		Ribonucleoprotein complex biogenesis (GO:0022613)
		Ribosomal small subunit biogenesis (GO:0042274)
	Downregulated	Purine ribonucleotide catabolic process (GO:0009154)
		Purine ribonucleotide metabolic process (GO:0009150)
		Protein geranylgeranylation (GO:0018344)
		Macromolecule modification (GO:0043412)
		UDP-N-acetylglucosamine biosynthetic process (GO:0006048)
		Protein prenylation (GO:0018342)
		Proteolysis (GO:0006508)
		Negative regulation of interferon-beta production (GO:0032688)
		UDP-N-acetylglucosamine metabolic process (GO:0006047)
		AMP metabolic process (GO:0046033)

On a structural level, as depicted in Table 5 (Supplementary Table S13), cellular components involved in cytoskeletal support and intercellular junctions—such as the actin cytoskeleton and adherens junctions—were enriched, reinforcing the concept that epithelial sheet migration during wound healing relies on the dynamic reassembly of intracellular scaffolds and adhesion complexes.

**TABLE 5:** Top 10 *GO Cellular Components* terms regulated similarly in human and mouse during corneal wound healing.

GO Analysis		*Term*
GO cellular components	Upregulated	Focal adhesion (GO:0005925)
		Cell-substrate junction (GO:0030055)
		Cytosolic large ribosomal subunit (GO:0022625)
		Large ribosomal subunit (GO:0015934)
		Cytosolic small ribosomal subunit (GO:0022627)
		Small ribosomal subunit (GO:0015935)
		Secretory granule lumen (GO:0034774)
		Intracellular organelle lumen (GO:0070013)
		Collagen-containing extracellular matrix (GO:0062023)
		Ribosome (GO:0005840)
	Downregulated	Vesicle (GO:0031982)
		Golgi membrane (GO:0000139)
		Ficolin-1-rich granule membrane (GO:0101003)
		Tertiary granule (GO:0070820)
		Secretory granule membrane (GO:0030667)
		Vesicle membrane (GO:0012506)
		Vacuolar lumen (GO:0005775)
		Ficolin-1-rich granule (GO:0101002)
		Specific granule lumen (GO:0035580)
		Bounding membrane of organelle (GO:0098588)

Protease activity, while essential for matrix remodeling and immune defense, must be tightly regulated in the cornea to avoid stromal degradation and opacity, as shown in Table 6 (Supplementary Table S14). In line with this, multiple peptidase functions were significantly downregulated, including “Aminopeptidase Activity,” “Cysteine-Type Peptidase Activity,” and “Calcium-Dependent Phospholipid Binding.” This suggests a conserved strategy to restrain excessive proteolysis during healing, likely protecting the extracellular matrix (ECM) from premature breakdown and preserving corneal transparency.

**TABLE 6:** Top 10 *GO Molecular function* terms regulated similarly in human and mouse during corneal wound healing.

GO Analysis		*Term*
GO molecular function	Upregulated	Cadherin binding (GO:0045296)
		mRNA binding (GO:0003729)
		Endopeptidase inhibitor activity (GO:0004866)
		Serine-type endopeptidase inhibitor activity (GO:0004867)
		Calcium ion binding (GO:0005509)
		Peptidase inhibitor activity (GO:0030414)
		Metal ion binding (GO:0046872)
		Lipoprotein particle receptor binding (GO:0070325)
		Actin binding (GO:0003779)
		mRNA 3′-UTR binding (GO:0003730)
	Downregulated	Calcium-dependent phospholipid binding (GO:0005544)
		Transition metal ion binding (GO:0046914)
		Exopeptidase activity (GO:0008238)
		Lysophospholipase activity (GO:0004622)
		Cysteine-type peptidase activity (GO:0008234)
		Endopeptidase activity (GO:0004175)
		ADP binding (GO:0043531)
		Aminopeptidase activity (GO:0004177)

Similarly, terms associated with vesicle-mediated secretion, such as “Golgi Membrane” and “Vesicle,” were downregulated, pointing to a temporary reprioritization away from constitutive secretion. While certain vesicle-related pathways (e.g., COPI trafficking) may be locally upregulated to supply healing factors, broader suppression of secretory machinery may reflect a resource-saving mechanism or a means to limit inflammation.

Despite the downregulation of glycosylation and certain biosynthetic pathways, the tear proteome revealed signs of upregulated protein synthesis, including the activation of “aminoacyl-tRNA ligase activity” and “5S rRNA binding.” This likely reflects the increased translational demands of epithelial cells undergoing proliferation and repair.

Simultaneously, metabolic shifts were evident. GO biological processes involved in amino sugar biosynthesis, glycerolipid catabolism, and AMP metabolism were downregulated, indicating a reallocation of metabolic resources from general cellular maintenance toward tissue regeneration. These changes align with the concept of a metabolic reboot typical of wound-healing tissues, in which cells temporarily divert energy and substrates to support critical repair pathways [19].

Altogether, the shared enrichment profile observed in both human and murine tear proteomes reveals a core response to corneal injury that integrates innate immune activation, cytoskeletal remodeling, suppression of excessive proteolysis, and a rewiring of metabolic priorities. However, less than 40% of the corneal injury response were conserved between human and mouse, therefore, we investigated the species-specificities of the tear film response to corneal injury.

### Species-specific molecular pathways shaping corneal wound healing dynamics

To uncover divergent mechanisms between humans and mice during corneal wound healing, we analyzed species-specific enrichment profiles derived from the tear proteome. Using Reactome and Gene Ontology annotations, we identified biological processes, cellular components, and molecular functions that were selectively enriched in either human or murine models. These findings reflect distinct physiological priorities and tissue responses, shedding light on both shared and unique aspects of the corneal healing trajectory in different organisms.

The human-specific Reactome enrichment terms revealed a strong emphasis on complement activation and secretory trafficking, as detailed in Table 7 (Supplementary Table S15). Key pathways included the “Complement Cascade,” “Intrinsic Pathway of Fibrin Clot Formation,” and “COPI-mediated Anterograde Transport,” alongside “Golgi-Associated Vesicle Biogenesis” and “RAB Geranylgeranylation.” This enrichment pattern indicates a coordinated mobilization of secretory and immune machinery in the human tear film. The tear proteome appears to serve not only as a reservoir of inflammatory mediators but also as a conduit for vesicle-mediated replenishment of protective and structural proteins essential for healing. Concordantly, human-specific GO Biological Processes were enriched in “Protein Localization,” “Positive Regulation of Cell-Substrate Adhesion,” and “Positive Regulation of Secretion by Cell,” emphasizing directed trafficking and epithelial migration, as reported in Table 8 (Supplementary Table S16). The upregulation of endothelial barrier formation and ERK/MAPK signaling cascades further supports the notion of a complex, tightly regulated epithelial repair program involving both secretory and migratory components. At the cellular level, human-enriched terms included “Cytoplasmic Vesicle Membrane,” “Very-Low-Density Lipoprotein Particles,” and “Membrane Attack Complex,” reflecting not only vesicular dynamics but also immune surveillance functions, as indicated in Table 9 (Supplementary Table S17). The presence of lipoprotein particle-related terms also suggests modulation of lipid-based components of the tear film, which are essential for ocular surface stability. Human-specific molecular functions highlighted an increase in “GTPase Activity,” “GDP Binding,” and “Myosin Binding,” which point to cytoskeletal control and vesicle trafficking via small GTPase signaling, as depicted in Table 10 (Supplementary Table S18). The actin-myosin axis appears especially prominent, likely supporting epithelial cell shape changes and dynamic migration.

**TABLE 7:** Top 5 species-specificities in *Reactome Pathways* regulation during corneal wound healing.

Species		*Term*	*p-value*	*q-value*
Human-specific reactome pathways	Upregulated	Regulation of complement cascade	1,85E-17	4,23E-16
		Complement cascade	2,47E-17	5,56E-16
		COPI-mediated anterograde transport	1,49E-11	3,06E-10
		Intrinsic pathway of fibrin clot formation	4,91E-11	9,91E-10
		Golgi associated vesicle biogenesis	2,74E-10	5,29E-09
	Downregulated	Scavenging of heme from plasma	5,40E-79	2,39E-76
		Classical antibody-mediated complement activation	3,67E-76	8,11E-74
		FCGR activation	4,63E-74	6,82E-72
		Role of LAT2 NTAL LAB on calcium mobilization	9,91E-74	1,09E-71
		Creation of C4 and C2 activators	2,09E-73	1,85E-71
Murine-specific reactome pathways	Upregulated	Glycosphingolipid catabolism	1,51E-06	2,87E-05
		Aerobic respiration and respiratory electron transport	2,97E-06	5,47E-05
		Beta oxidation of hexanoyl-CoA to butanoyl-CoA	3,64E-06	6,42E-05
		Beta oxidation of lauroyl-CoA to decanoyl-CoA-CoA	3,64E-06	6,42E-05
		Beta oxidation of octanoyl-CoA to hexanoyl-CoA	3,64E-06	6,42E-05
	Downregulated	Membrane trafficking	2,23E-54	2,63E-51
		Golgi-to-ER retrograde transport	2,32E-23	4,56E-21
		ER to golgi anterograde transport	3,71E-23	6,23E-21
		Cellular responses to stimuli	1,09E-22	1,61E-20
		MHC class II antigen presentation	2,59E-22	3,15E-20

**TABLE 8:** Top 5 species-specificities in *GO Biological Process* regulation during corneal wound healing.

Species		*Term*	*p-value*	*q-value*
Human-specific GO biological process	Upregulated	Protein transport (GO:0015031)	3,60E-19	1,65E-16
		Protein localization (GO:0008104)	4,73E-12	9,28E-10
		Positive regulation of cell-substrate adhesion (GO:0010811)	1,98E-10	2,86E-08
		Intracellular protein transport (GO:0006886)	3,27E-10	4,50E-08
		Positive regulation of secretion by cell (GO:1903532)	1,57E-07	1,60E-05
	Downregulated	Protein glycosylation (GO:0006486)	1,10E-05	1,03E-02
		Purine ribonucleoside monophosphate catabolic process (GO:0009169)	5,91E-05	1,64E-02
		Protein O-linked glycosylation (GO:0006493)	1,19E-04	1,71E-02
		Protein N-linked glycosylation (GO:0006487)	1,23E-04	1,71E-02
		Oligosaccharide biosynthetic process (GO:0009312)	1,29E-04	1,71E-02
Murine-specific GO biological process	Upregulated	N-glycan processing (GO:0006491)	1,50E-06	1,72E-04
		GMP biosynthetic process (GO:0006177)	1,07E-05	7,79E-04
		Nuclear envelope organization (GO:0006998)	1,24E-05	8,57E-04
		Protein localization to nucleus (GO:0034504)	2,31E-05	1,52E-03
		IMP biosynthetic process (GO:0006188)	2,43E-05	1,56E-03
	Downregulated	Protein-containing complex assembly (GO:0065003)	2,35E-13	2,97E-10
		Protein localization (GO:0008104)	5,03E-12	4,24E-09
		Endoplasmic reticulum to golgi vesicle-mediated transport (GO:0006888)	7,23E-12	4,57E-09
		Intracellular protein transport (GO:0006886)	1,05E-10	5,31E-08
		Membrane organization (GO:0061024)	2,93E-10	1,06E-07

**TABLE 9:** Top 5 species-specificities in *GO Cellular Components* regulation during corneal wound healing.

Species		*Term*	*p-value*	*q-value*
Human-specific GO cellular components	Upregulated	Triglyceride-rich plasma lipoprotein particle (GO:0034385)	2,02E-10	2,32E-09
		Very-low-density lipoprotein particle (GO:0034361)	2,02E-10	2,32E-09
		Cytoplasmic vesicle membrane (GO:0030659)	5,90E-10	6,52E-09
		Platelet dense granule (GO:0042827)	8,01E-08	6,21E-07
		Membrane attack complex (GO:0005579)	2,37E-07	1,62E-06
	Downregulated	Lysosomal lumen (GO:0043202)	6,19E-04	1,87E-02
		Golgi medial cisterna (GO:0005797)	4,96E-03	7,80E-02
		Extracellular vesicle (GO:1903561)	6,74E-03	9,27E-02
		Azurophil granule membrane (GO:0035577)	3,32E-02	2,72E-01
		Microvillus (GO:0005902)	3,32E-02	2,72E-01
Murine-specific GO cellular components	Upregulated	Mitochondrial matrix (GO:0005759)	8,11E-05	7,35E-04
		Hemidesmosome (GO:0030056)	4,76E-04	3,50E-03
		Mitochondrial inner membrane (GO:0005743)	5,11E-03	2,68E-02
		Mitochondrial membrane (GO:0031966)	5,82E-03	2,93E-02
		Pyruvate dehydrogenase complex (GO:0045254)	1,20E-02	5,35E-02
	Downregulated	Ficolin-1-rich granule lumen (GO:1904813)	1,77E-18	2,12E-16
		Secretory granule lumen (GO:0034774)	3,50E-17	2,79E-15
		Cytoplasmic vesicle lumen (GO:0060205)	4,17E-16	2,49E-14
		Focal adhesion (GO:0005925)	4,98E-12	2,38E-10
		Cell-substrate junction (GO:0030055)	9,72E-12	3,87E-10

**TABLE 10:** Top 5 species-specificities in *GO Molecular Function* regulation during corneal wound healing.

Species		*Term*	*p-value*	*q-value*
Human-specific GO molecular function	Upregulated	Ribonucleoside triphosphate phosphatase activity (GO:0017111)	4,55E-13	4,89E-11
		GTPase activity (GO:0003924)	8,85E-13	7,61E-11
		GDP binding (GO:0019003)	3,55E-10	2,18E-08
		Guanyl ribonucleotide binding (GO:0032561)	1,79E-08	9,62E-07
		GTP binding (GO:0005525)	4,29E-07	1,23E-05
	Downregulated	Nucleotidase activity (GO:0008252)	1,90E-05	3,98E-03
		5′-nucleotidase activity (GO:0008253)	3,55E-04	3,71E-02
		Pyrophosphatase activity (GO:0016462)	7,32E-04	5,10E-02
		Protein geranylgeranyltransferase activity (GO:0004661)	1,42E-03	7,44E-02
		Beta-galactoside (CMP) alpha-2,3-sialyltransferase activity (GO:0003836)	2,12E-03	7,59E-02
Murine-specific GO molecular function	Upregulated	Hydroxymethyl-, formyl- and related transferase activity (GO:0016742)	2,43E-05	1,51E-03
		Phosphoric diester hydrolase activity (GO:0008081)	3,75E-05	2,07E-03
		Oxidoreductase activity, acting on the CH-OH group of donors, NAD or NADP as acceptor (GO:0016616)	2,06E-04	8,06E-03
		Phospholipase D activity (GO:0004630)	2,43E-04	8,06E-03
		3-Hydroxyacyl-CoA dehydrogenase activity (GO:0003857)	2,43E-04	8,06E-03
	Downregulated	Cadherin binding (GO:0045296)	4,88E-32	2,27E-29
		Anion binding (GO:0043168)	6,40E-11	1,49E-08
		Protein homodimerization activity (GO:0042803)	9,93E-11	1,54E-08
		Protein kinase binding (GO:0019901)	1,17E-06	1,36E-04
		Intramolecular phosphotransferase activity (GO:0016868)	1,71E-06	1,47E-04

Together, these results suggest that the human cornea relies on a secretory-driven and immunologically alert environment, with a heavy reliance on vesicle-mediated trafficking and barrier integrity maintenance. This response likely reflects evolutionary pressure to protect a larger, longer-lived, and exposed corneal surface from both microbial invasion and physical desiccation.

In contrast, murine-specific enrichment profiles were dominated by mitochondrial and metabolic processes. Reactome terms such as “Aerobic Respiration and Respiratory Electron Transport,” and various “Beta Oxidation” pathways revealed a pronounced shift toward oxidative energy production and lipid metabolism, as shown in Table 7 (Supplementary Table S15). These pathways likely support the intense energy demands of rapid re-epithelialization, a hallmark of the murine corneal response. GO Biological Processes enriched in the murine tear proteome included “GMP Biosynthetic Process,” “Nuclear Envelope Organization,” and “Unfolded Protein Response,” consistent with a metabolically activated and stress-adapted cellular state, as detailed in Table 8 (Supplementary Table S16). Upregulation of nucleotide biosynthesis suggests preparation for proliferation and DNA replication, while nuclear reorganization and UPR activation indicate mechanisms for coping with translational load and oxidative stress. At the structural level, murine-enriched GO Cellular Components centered on mitochondria: “Mitochondrial Inner Membrane,” “Pyruvate Dehydrogenase Complex,” and “Alpha-Ketoacid Dehydrogenase Complex” all point toward active mitochondrial energy metabolism, as reported in Table 9 (Supplementary Table S17). In parallel, enrichment of “Hemidesmosomes” underscores the reinforcement of epithelial–basement membrane anchoring, stabilizing the newly formed epithelium during high-turnover repair. Molecular functions further reinforce this metabolic theme, as presented in Table 10 (Supplementary Table S18). Enriched murine-specific activities included “Oxidoreductase Activity,” “Phospholipase D Activity,” and “3-Hydroxyacyl-CoA Dehydrogenase Activity,” which contribute to lipid breakdown, redox balance, and mitochondrial fatty acid metabolism. These processes are critical for fueling the epithelial regenerative process and maintaining homeostasis under injury-induced oxidative stress.

Collectively, the murine corneal healing response appears to emphasize rapid, energy-intensive, and metabolically efficient tissue regeneration, supported by mitochondrial activation and reduced reliance on external secretory inputs. This contrasts with the human reliance on tear-based immune and secretory support and reflects species-specific strategies shaped by corneal size, life span, and immune architecture.

### Conserved secreted tear proteins staging injury and resolution

While these datasets frame the corneal wound-healing landscape, we leaveraged the murine and human tear proteome to identify putative secreted biomarkers by using a stringent filter. We selected secreted proteins exhibiting p < 0.001 and FC >2.75 to identify high-confidence biomarker candidates, in order to prioritise large-effect, highly significant proteins and thus improve specificity and robustness of the final biomarker panel (Supplementary Tables S19, 20). Across human PRK and murine epithelial abrasion, we identify a compact cross-species tear panel that rises early after injury and recedes during resolution, as shown in Table 11 (Supplementary Figure S1): transferrin (TF) and hemopexin (HPX) index iron/heme scavenging; albumin (ALB) reflects acute vascular leak; apolipoprotein A-I (APOA1) tracks lipid transport for barrier repair; and the coagulation modulators kininogen-1 (KNG1) and α2-antiplasmin (SERPINF2) report protease/fibrinolysis control. These proteins increase consistently at the earliest time points (human D0; mouse 6–12 h) and trend toward baseline during recovery (human D3; mouse ∼24 h), providing a shared kinetic signature of acute epithelial injury and restitution. Focusing on this small, conserved set emphasizes translational feasibility: the same markers can stage wound response in preclinical models and in patients. While additional human- or mouse-preferential signals exist, as displayed in Table 12, the TF/HPX/ALB/APOA1/KNG1/SERPINF2 core captures the dominant, conserved biology and supports standardized sampling at D0 and D3 in humans (6–24 h in mice) to quantify peak and early resolution phases with minimal assays.

**TABLE 11:** List of all biomarkers, selected through stringent filter (FC >2.75 and p-val <0.001), common to the human and murine corneal wound healing response.

Common proteins	Murine				Human	
	Fold change T6 vs. CTRL	Fold change T12 vs. CTRL	Fold change T18 vs. CTRL	Fold change T24 vs. CTRL	Fold change D0 vs. Pre	Fold change D3 vs. Pre
Gc	27,92	22,52	8,74	6,30	3,74	1,16
Tf	25,55	21,10	9,10	6,20	9,68	1,22
Alb	24,27	20,10	11,26	8,55	8,20	1,25
Ttr	24,26	17,88	9,52	6,48	4,33	1,08
Apoa4	24,25	10,52	8,89	7,80	6,54	0,93
Hpx	22,91	20,77	9,36	6,37	14,88	1,32
Kng1	22,10	18,56	9,04	6,54	4,59	1,12
Serpinf2	18,43	17,23	9,70	6,90	5,76	1,17
Ahsg	9,73	18,41	11,43	8,50	6,69	1,12
Apoa1	9,67	10,26	9,32	7,84	21,80	1,09
Serpinc1	9,15	8,60	4,03	3,00	5,45	1,21
A1bg	5,30	6,18	3,73	2,83	4,76	1,12
Agt	3,43	6,20	5,10	5,51	4,40	1,23
Itih2	1,46	2,84	2,42	2,17	11,88	1,59

**TABLE 12:** Top10 of species-specific biomarkers, selected through stringent filter (FC >2.75 and p-val <0.001), involved in corneal wound healing response.

Species	Protein	Fold change max
Human-specific biomarkers	CGA	20,48
	TGFBI	50,16
	LUM	34,65
	C8B	19,77
	A2M	17,36
	IGHG2	16,40
	PTGDS	16,38
	FGG	15,41
	IGHG3	15,32
	KERA	15,17
Murine-specific biomarkers	Serpina3k	26,80
	Serpina1c	26,19
	Serpina1b	18,32
	Serpina3n	10,31
	C3	10,50
	Cfd	8,26
	Fetub	7,43
	Ighg2b	7,53
	Azgp1	6,22
	Cp	7,11

## Discussion

The present meta-analysis of tear proteomic dynamics during corneal wound healing reveals an evolutionarily conserved yet distinctly modulated biological response across species. By comparing murine and human datasets [15, 16], we identified not only shared molecular mediators underpinning the core wound-healing program, but also species-specific adaptations that reflect divergent physiological priorities. These results offer both mechanistic insights into corneal regeneration and a rational basis for developing targeted therapeutic strategies adapted to human biology.

Our initial observation was a notable difference in the number of proteins identified: 2,025 in human tear film versus 3,618 in murine samples. This marks the most extensive murine tear proteome reported in the literature so far, and comes reasonably close to the highest number of proteins identified in human tears (>3,000) [20]. This discrepancy likely stems from differences in sampling methodologies. Human tears were collected using Schirmer strips, whereas murine tears were obtained via glass microcapillary. Previous studies have demonstrated that while capillary collection offers superior reproducibility compared to Schirmer strips, it generally yields a lower protein concentration and reduced diversity [21]. Consequently, the higher number of proteins detected in murine samples suggests that the murine tear film may possess intrinsically greater proteomic complexity.

At the core of the conserved response is a tightly regulated interplay between innate immunity, cytoskeletal remodeling, proteolysis suppression, and metabolic reprioritization. The early upregulation of complement activation pathways, particularly the alternative pathway (C3/C5) and terminal lytic components, emphasizes the tear film role as a frontline immune barrier. This innate immune tightly regulated activation, that likely integrates complement activation with Toll-like receptor signalling [22], provides rapid antimicrobial defense, neutralizing pathogens introduced through corneal abrasion or surgery. Simultaneously, the suppression of adaptive immune signatures such as MHC class II antigen presentation and Fc receptor signaling suggests a protective strategy to minimize inflammation-driven tissue damage. This dichotomy—immune activation without overactivation—may be especially critical for preserving corneal transparency. These findings are consistent with the view of the ocular surface as a mucosal barrier in which the tear film functions as a secretory–immune surveillance interface. Lacrimal and accessory glands continuously deliver antimicrobial proteins, complement factors, immunoglobulins and cytokines into the tear film to provide rapid pathogen sensing and neutralisation while preserving epithelial integrity and immune tolerance [23, 24]. In parallel, tear-derived extracellular vesicles act as mobile carriers of immune and stress signals whose cargo reflects the state of ocular surface epithelia and immune cells, and are increasingly recognised as diagnostic and therapeutic tools in ocular and neurodegenerative disease [25, 26].

Parallel to immune regulation, our analyses uncovered the robust activation of cytoskeletal pathways, including actin filament organization, actomyosin contractility, and cadherin-mediated adhesion. These pathways are essential for the collective migration of epithelial cells that reseal the wounded surface. Structural components such as adherens junctions and the actin cytoskeleton were strongly enriched, supporting the idea that mechanical integrity and dynamic motility are orchestrated in concert. Notably, this remodeling appears coordinated with increased protein synthesis, as evidenced by the upregulation of tRNA ligase activity and ribosomal binding functions—molecular hallmarks of heightened cellular activity during tissue regeneration.

The regulation of proteolytic activity emerged as another conserved axis. While certain matrix-degrading enzymes may assist in clearing damaged ECM and facilitating migration, our data show consistent downregulation of cysteine-type and aminopeptidase activities. Such restraint is likely vital for protecting stromal collagen from degradation and maintaining the biomechanical integrity of the cornea. In addition, suppression of vesicle-mediated secretion and glycosylation pathways suggests a temporary downshifting of secretory processes, perhaps to reduce metabolic load or avoid excessive extracellular signaling, nderscoring that controlling extracellular protease activity is central to scar-free corneal repair [27].

Importantly, our findings also highlight species-specific differences that must be carefully considered when translating preclinical data to human contexts. In humans, the tear film response is characterized by pronounced secretory and immune surveillance functions. Complement activation, vesicle trafficking (COPI/Golgi), and endothelial barrier maintenance are central components. These likely reflect both the architectural complexity of the human cornea and its prolonged exposure to environmental insults. The reliance on secretory machinery may represent an evolved adaptation to sustain ocular surface integrity over a longer lifespan and within a more variable ecological niche.

By contrast, the murine cornea responds with a metabolic reboot marked by mitochondrial activation, β-oxidation, and nucleotide biosynthesis. These features support a highly energy-demanding regenerative process, consistent with the rapid epithelial turnover observed in rodents. Furthermore, the enrichment of nuclear envelope reorganization and unfolded protein response pathways underscores the need for cellular resilience during high-throughput protein production. The murine corneal epithelium appears optimized for rapid restoration rather than long-term maintenance, a distinction likely shaped by differences in size, ocular exposure, and immune architecture. This metabolic and mitochondrial program is in line with the broader concept that successful tissue repair requires a stage-specific “metabolic orchestration” of wound healing, involving coordinated shifts between glycolysis, oxidative phosphorylation and lipid metabolism [28]. Recent work in the cornea further supports a central role for mitochondrial homeostasis in epithelial regeneration: CISD2-dependent control of Ca^2+^ and mitochondrial integrity promotes corneal epithelial renewal, and defects in mitochondrial dynamics impair epithelial healing and contribute to neurotrophic keratopathy [29, 30]. Conversely, transplantation of healthy mitochondria into injured corneal epithelium accelerates wound closure and restores barrier function [31]. Our proteomic “metabolic reboot” signature likely captures this conserved mitochondrial–metabolic reset that enables rapid proliferation and migration of corneal epithelial cells after PRK-like injury.

We speculate that these divergent programmes reflect distinct evolutionary constraints on the human versus murine ocular surface. In humans, long lifespan, continuous daytime visual demand and decades of exposure to desiccating stress, pollutants and pathogens favour a strategy built around tear-film–driven secretion, immune surveillance and tightly regulated mucosal tolerance at the ocular surface [24, 32]. This is consistent with the concept of the ocular surface as a specialised mucosal immune organ in which lacrimal and conjunctival secretions, goblet cell function and local regulatory circuits jointly maintain barrier integrity while minimising bystander damage to a transparent tissue that must remain optically clear for many decades [24, 32]. In contrast, the murine corneal epithelium is a rapidly renewing, limbal stem cell–driven barrier capable of restoring coverage within hours to days [33], making a fast, metabolically intensive “reboot” of epithelial proliferation and mitochondrial activity a plausible priority for a short-lived, small mammal. Comparative immunology further indicates that murine and human immune systems have evolved under different selective pressures, with distinct balances of innate and adaptive effector strategies and tolerance mechanisms [34, 35]. Together, these factors may bias humans towards a chronic, secretion- and surveillance-centred response to epithelial injury, whereas mice rely more heavily on a transient metabolic surge to rapidly re-establish epithelial coverage.

These divergent signatures underscore the limitations of directly extrapolating murine data to human therapeutics. While the murine model remains invaluable for delineating fundamental biological processes, its metabolism-driven healing profile may favor therapeutic strategies aimed at enhancing energy metabolism or epithelial proliferation. In contrast, the human tear film response relies more heavily on immune modulation and secretory activity, implying that interventions promoting vesicle trafficking, immune surveillance, or epithelial barrier stabilization may be more effective in clinical settings.

Importantly, our identification of conserved and species-specific tear film pathways carries strong implications for translational research. First, the delineation of shared molecular responses opens new avenues for biomarker discovery, providing researchers with a molecular blueprint to monitor corneal healing across species. These shared pathways offer a robust foundation for identifying cross-species diagnostic or prognostic markers, thereby facilitating the evaluation of therapeutic efficacy in both preclinical and clinical studies, and the importance of human-specific tear signatures for translational biomarker development [11, 12].

To enhance the robustness of our biomarker screening, we applied stricter criteria by combining a lowed p-adj threshold (<0.001) and a higher fold change cutoff (2.75), as previously done [16, 36]. By increasing the stringency and the biological relevance of our findings in the proteomics datasets, we focused on the most compelling and physiologically relevant candidate biomarkers. Thus, beyond pathway-level concordance, our cross-species analysis distilled a minimal set of secreted tear proteins that stage the epithelial response and its early resolution—transferrin and hemopexin (iron/heme scavenging), albumin (vascular leak), apolipoprotein A-I (lipid transport/barrier support), and the coagulation modulators kininogen-1 and α2-antiplasmin (protease/fibrinolysis control). This conserved panel increases at the first post-injury sampling (human D0; mouse 6–12 h) and trends back toward baseline during recovery (human D3; mouse ∼24 h), providing a shared kinetic signature that is both mechanistically interpretable and operationally simple. Because the same readouts are measurable in mice and in patients, they offer practical pharmacodynamic markers for interventions aimed at tightening barrier function, limiting hemoprotein toxicity, and rebalancing protease activity, and they support standardized sampling windows (D0/D3 in humans; 6–24 h in mice). Incorporating this compact panel alongside pathway enrichments strengthens the translational bridge from preclinical benchmarking to early clinical decision-making.

Second, our work provides critical insight for drug development pipelines that aim to leverage tears as a minimally invasive “liquid biopsy” of the ocular surface. Therapeutics often move from murine models to human applications, yet the failure to account for species-specific wound healing responses can compromise translational success. Here, our abrasion mouse model is a valuable strategy to study corneal epithelial wound healing, but does not fully replicate the PRK procedure in patients, as the stroma is also reshaped with a laser after the epithelial removal during the refractive surgery. By clearly mapping which pathways are conserved and which diverge between mice and humans, our study offers a strategic guide for anticipating efficacy, optimizing drug targets, and minimizing translational failure. Pharmaceutical developers can now design and test compounds with a clearer understanding of the biological context in which they will operate, selecting therapeutic windows that are biologically relevant across species or tailoring strategies to human-specific repair mechanisms, and aligns with current efforts to exploit tear proteomics for therapeutic stratification and monitoring [17, 18, 37].

The methodological disparity between the human and mouse models represents a limitation of this study. Mouse corneal scraping does not fully mimic the tissue alterations associated with human PRK, which involves both epithelial abrasion and stromal laser ablation. These differences may influence wound healing dynamics and could contribute to species-specific variations in tear protein profiles, as stronger matrix reactions may affect the human proteome. Moreover, the tear sampling method differs between patients and mice, which are respectively collected using Schirmer strips and microcapillary glass tubes. This may affect protein recovery and composition, contributing to differences in the proteomic profile, introducing an additional source of variability. Although these factors do not undermine the overall comparisons, they represent intrinsic limitations of the study design and should be taken into consideration when analysis the data.

In conclusion, this study presents the first integrative, cross-species analysis of tear proteomic signatures during corneal wound healing. It defines a conserved biological core essential for epithelial regeneration and reveals evolutionary adaptations that fine-tune the repair strategy to species-specific constraints. These insights not only deepen our understanding of ocular surface biology but also provide a valuable framework for biomarker identification and therapeutic translation, offering a pathway toward precision medicine approaches that improve patient outcomes in corneal disease.

## Data Availability

The mass spectrometry proteomics datasets are available in the PRIDE repository, via ProteomeXchange, with the identifier PXD061492 (mouse) and PXD067691 (human).

## Footnotes

1
https://maayanlab.cloud/Enrichr/

2
https://appyters.maayanlab.cloud/
