# Supplementary figures and images for: Integrated tear proteomics define the molecular blueprint of corneal epithelial repair
Source: Exp Biol Med (Maywood). 2026 Feb 2;250:10866. doi: 10.3389/ebm.2025.10866 (PMC12908170; doi:10.3389/ebm.2025.10866)

FIGURE 1

**a**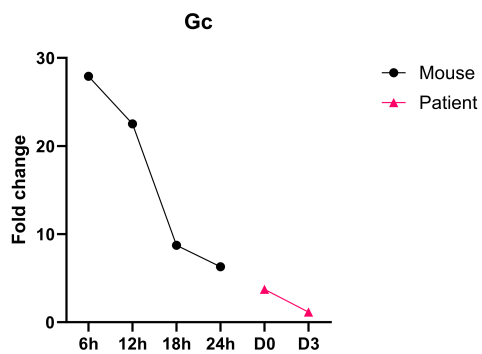**b**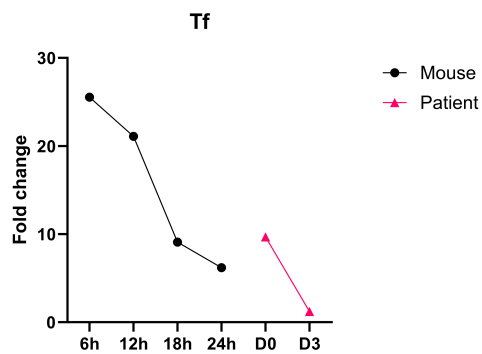**c**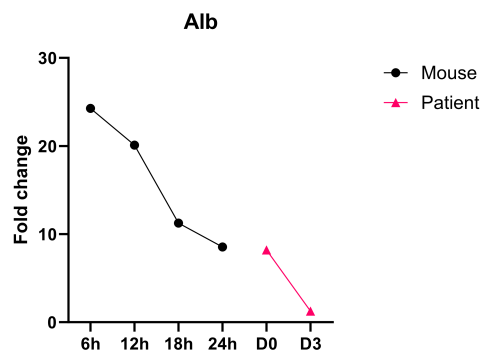**d**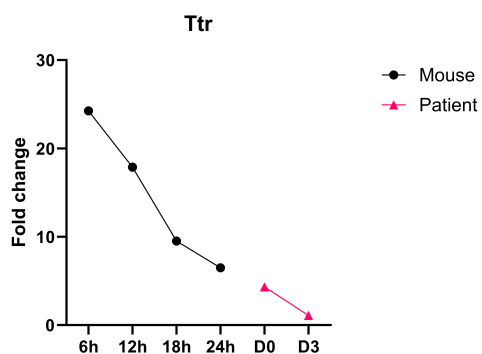**e**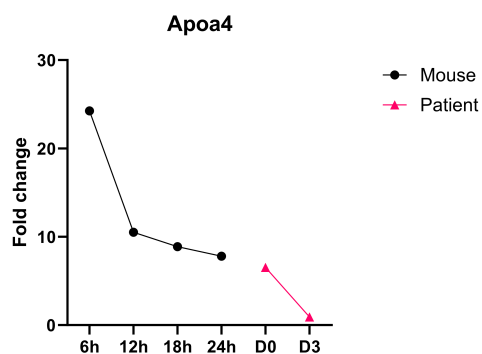**f**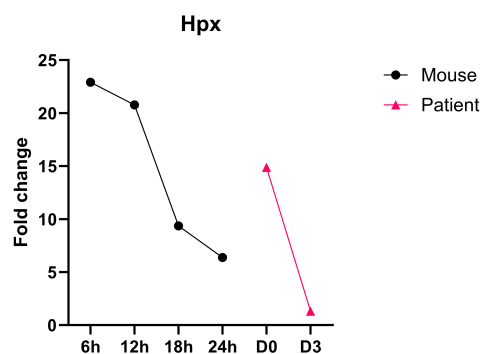**g**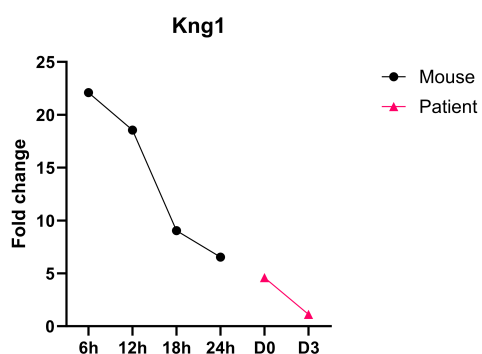**h**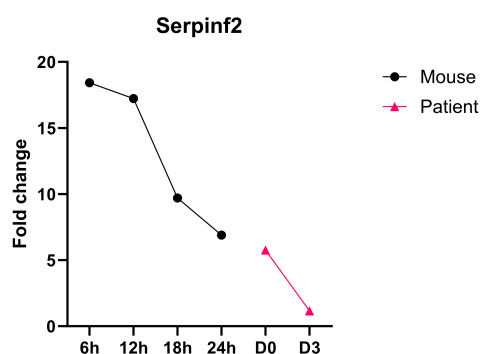**i**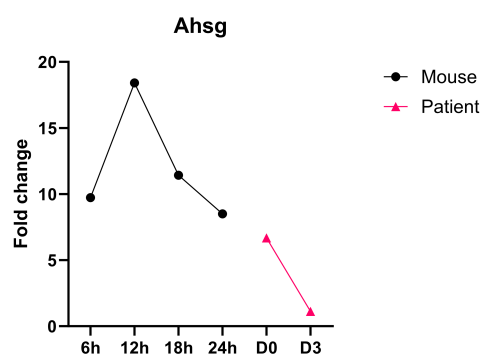**j**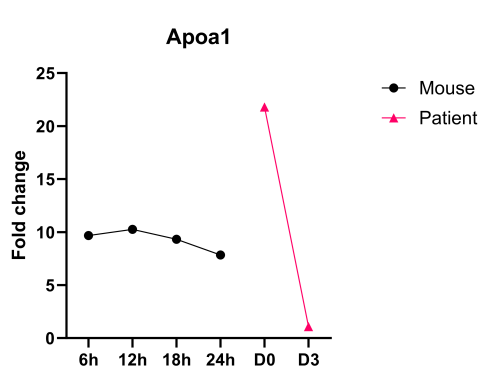**k**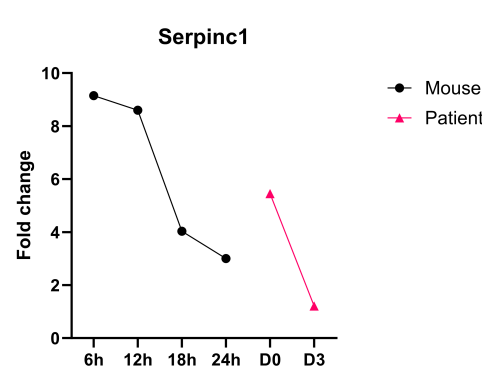**l**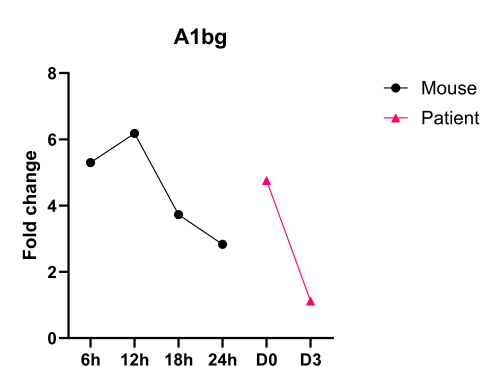**m**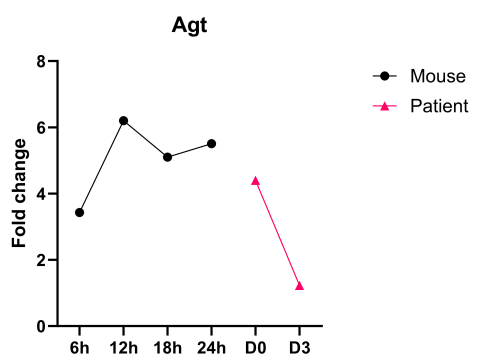**n**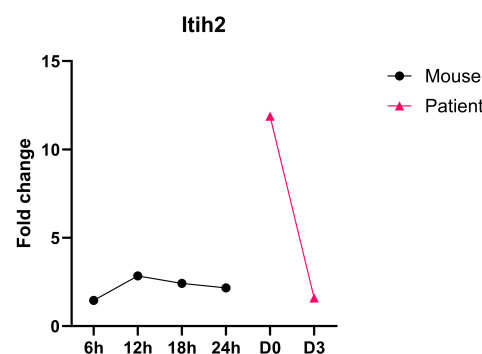

Supplement: Supplementary file 1 [file DataSheet1.pdf]
