# Supplementary material for: Integrated tear proteomics define the molecular blueprint of corneal epithelial repair
Source: Exp Biol Med (Maywood). 2026 Feb 2;250:10866. doi: 10.3389/ebm.2025.10866 (PMC12908170; doi:10.3389/ebm.2025.10866)
Supplement: Supplementary file 2 [file Supplementaryfile2.docx]

**Supplementary Material**

**Figure S1.** Graphical representations of biomarkers fold change evolution during corneal wound healing. Murine fold changes are in black (6h, 12h, 18h, 24h compared to “before abrasion”), patients fold changes are in red (D0, D3 compared to “before PRK”).

**Table S1.** Lists of all proteins identified in the murine tear film during corneal wound healing.

**Table S2.** Lists of all proteins identified in the human tear film during corneal wound healing.

**Table S3.** Lists of all proteins up and downregulated in the murine and human tear film during corneal wound healing, in alphabetical order.

**Table S4.** Lists of the proteins that are similarly up and downregulated in the murine and human tear film during corneal wound healing, in alphabetical order.

**Table S5.** List of all the *Reactome Pathways* terms obtained from the proteins that are upregulated similarly in murine and human tear film during corneal wound healing.

**Table S6.** List of all the *GO Biological Process* terms obtained from the proteins that are upregulated similarly in murine and human tear film during corneal wound healing.

**Table S7.** List of all the *GO Molecular Function* terms obtained from the proteins that are upregulated similarly in murine and human tear film during corneal wound healing.

**Table S8.** List of all the *Reactome Pathways* terms obtained from the proteins that are downregulated similarly in murine and human tear film during corneal wound healing.

**Table S9.** List of all the *GO Biological Process* terms obtained from the proteins that are downregulated similarly in murine and human tear film during corneal wound healing.

**Table S10.** List of all the *GO Molecular Function* terms obtained from the proteins that are upregulated similarly in murine and human tear film during corneal wound healing.

**Table S11.** List of all *Reactome Pathways* terms similarly regulated in human and murine tear film, in alphabetical order.

**Table S12.** List of all *GO Biological Process* terms similarly regulated in human and murine tear film, in alphabetical order.

**Table S13.** List of all *GO Cellular Components* terms similarly regulated in human and murine tear film, in alphabetical order.

**Table S14.** List of all *GO Molecular Function* terms similarly regulated in human and murine tear film, in alphabetical order.

**Table S15.** List of all species-specific *Reactome Pathways* terms regulated in human or murine tear film.

**Table S16.** List of all species-specific *GO Biological Process* terms regulated in human or murine tear film.

**Table S17.** List of all species-specific *GO Cellular Components* terms regulated in human or murine tear film.

**Table S18.** List of all species-specific *GO Molecular Function* terms regulated in human or murine tear film.

**Table S19.** List of all biomarkers, selected through stringent filter (FC>2.75 and p-val<0.001), specific to human corneal wound healing.

**Table S20.** List of all biomarkers, selected through stringent filter (FC>2.75 and p-val<0.001), specific to murine corneal wound healing.
